# Molecular characterization of the new clinical entity associated with congenital adrenal hyperplasia: the CAH-X syndrome in the Spanish population

**DOI:** 10.1515/almed-2023-0071

**Published:** 2023-08-25

**Authors:** Laura Martínez Figueras, Rafael Muñoz Pacheco, Dolores García González, María Arriba Domènech, Begoña Ezquieta Zubicaray

**Affiliations:** Instituto de Investigación Sanitaria Gregorio Marañón, Madrid, Spain; Laboratorio de Diagnóstico Molecular, Servicio de Bioquímica Clínica, Hospital General Universitario Gregorio Marañón, Madrid, Spain

**Keywords:** CAH-X syndrome, congenital adrenal hyperplasia, *CYP21A2*, hypermobility-type Ehlers–Danlos syndrome, tenascin, *TNXB*

## Abstract

**Objectives:**

The chimeras causing the CAH-X syndrome (SCAH-X) result from recombination between *CYP21A2*-*TNXB* and their respective pseudogenes (*CYP21A1P*-*TNXA*). The clinical manifestations of this syndrome include congenital adrenal hyperplasia (CAH) and Ehlers–Danlos syndrome (EDS). Since SCAH-X has been recently described, the number of publications available is limited. The objective of this study was to set up a molecular approach and a screening algorithm for detecting CAH-X chimeras, determine their frequency and distribution in the Spanish population, and assess their clinical pattern of occurrence in a group of patients.

**Methods:**

A total of 186 patients were eligible for CAH-X molecular genetic testing. Testing included MLPA, heterodimer detection by capillary gel electrophoresis, and sequencing of exons 40, 41, and 43 of *TNXB*. A review was performed of the medical history of 20 patients from three hospitals of reference and the signs and symptoms of EDS they exhibited.

**Results:**

In total, 78 CAH patients were carriers of CAH-X chimeras (41.9 %). Forty-six patients were carriers of CH1 (24.7 %), 24 of CH2 (12.9 %), and 8 of CH3 (4.3 %), with a heterogeneous geographical distribution. Seven (35 %) of the 20 carriers of a CAH-X chimera who underwent clinical examination experienced clinical manifestations of EDS.

**Conclusions:**

The impact of SCAH-X in the Spanish population was assessed by genetic testing. In the light of the clinical pattern of occurrence and significant prevalence of SCAH-X in the Spanish population, early diagnosis of this entity is essential for an appropriate follow-up of clinical manifestations.

## Introduction

Congenital adrenal hyperplasia (CAH) embraces a group of autosomal recessive disorders (biallelic entities) that result from a deficiency in some of the enzymes or proteins involved in the adrenal biosynthesis of cortisol and/or aldosterone (OMIM #201910). As many as 95 % of CAH patients have 21-OH (21-OHD) deficiency resulting from the presence of variants in the *CYP21A2* gene [1], [2], [3]. CAH is categorized into two clinical forms, as a function of the level of impairment of 21-OHenzyme activity [1], [2], [3]: classic or severe neonatal forms and nonclassic forms. The classic forms (CL: salt-wasting form, SW; simple virilizing form, SV) affect 1:12,000–1:14,000 live births [4, 5]. Patients with the classic forms exhibit severe biallelic alterations (homozygosity or compound heterozygosity) and are characterized by the virilization of external female genitalia, together with adrenal insufficiency associated or not with salt wasting. Nonclassic forms (NC) affect 1:100–1:1,000 live births [4] and, although they may be oligo- or even asymptomatic, they may also be associated with a severe abnormality in one of their alleles.

**Figure 1:**
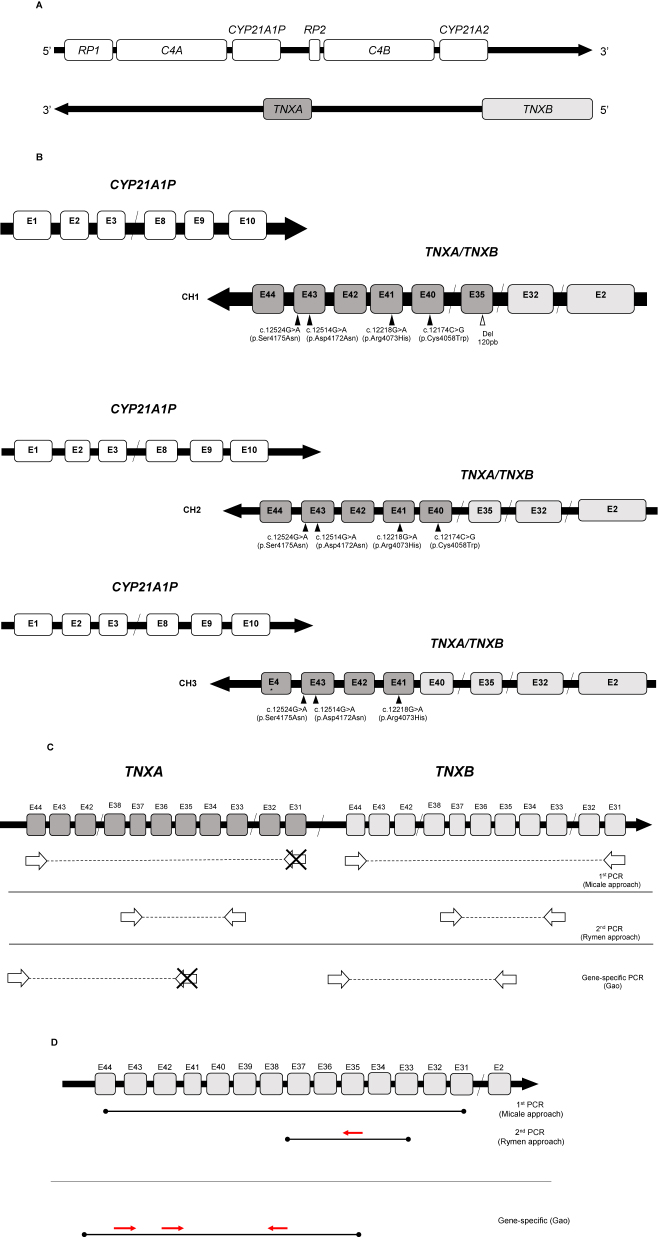
Genomic organization of locus 6p21.3, CAH-X chimeras and their molecular characterization. (A) Locus 6p21.3 and RCCX module showing the genes and respective pseudogenes (Adaptation from Marino, 2021). (B) Representation of the three types of CAH-X chimeras (CH1, CH2, and CH3) identified to date. The squares in clear gray represent *TNXB* exons, whereas *TNXA* exons are represented in dark gray. The molecular variants that characterize each chimera are indicated (Adaptation from Marino, 2021). (C) Scheme displaying the different techniques used for *TNXB* exon amplification for SCAH-X molecular characterization. The amplification primers used in PCRs are indicated next to the publication where they were suggested (Adaptation from Marino, 2021). (D) Scheme of the amplicons obtained by PCR and primers used for the sequencing of exons 35, 40, 41, and 43 of *TNXB*.

The *CYP21A2* gene is located in region III of the HLA system (6p21.3, human leukocyte antigen) [6]. Next to it, at a distance of 30kb [7], we find its nonfunctional pseudogene, *CYP21A1P* [8, 9]. The two genes share a 98 and 95 % homology in exons and introns, respectively [10]. This region is characterized by a particular structure that includes several pairs of genes/pseudogenes arranged in tandem, which makes of it a complex, difficult-to-sequence region [11]. These pairs include *RP1*, *C4A*, and *TNXB*, and their respective pseudogenes, *RP2*, *C4B*, and *TNXA*, which are not functional [11, 12] (Figure 1A). *TNXB* and *TNXA* are encoded on the complementary strand of *CYP21A2* and *CYP21A1P* and partially overlap with them in their 3′-UTR regions [13]. Whereas *TNXB* is a functional 44-exon gene, *TNXA* is truncated (4.5 kb) and shares homology with *TNXB* in exons 32–44 [13].

Tenascin (TNX), encoded by *TNXB*, is a protein of the extracellular matrix that belongs to the tenascin family. This glycoprotein expresses actively in connective tissue [14, 15], where it plays a structural role. TNX supports the macromolecular complexes of the matrix and collagen and elastin fibrils, thereby providing connective tissue with the appropriate biomechanical properties.

Monoallelic mutations (dominant pattern) in *TNXB* are associated with hypermobile Ehlers–Danlos syndrome (EDSh) (*International EDS Consortium*) [16]. EDS is a heterogeneous group of hereditary connective tissue disorders characterized by joint hypermobility, hyperextensibility, and skin fragility [17]. These syndromes have a prevalence of 1:5,000 in the general population [18] and are caused by a broad range of genetic mutations that frequently involve collagen metabolism [19].

The high homology between *CYP21A2* and *CYP21A1P* and between *TNXB* and *TNXA* together with their arrangement in tandem facilitate asymmetric pairing. This may result in conversion and rearrangement events, which give rise to the molecular alterations detected in this region. As a function of the location of the junction site, different hybrids or chimeras result, also known as *CYP21A2* “30 kb deletions.” (1) When asymmetric recombination has an intragenic junction site in *CYP21A2*, only this functional gene will be involved [12]. (2) When asymmetric recombination has a 3′- or postgenic junction site, the resulting deletion hybrids may also involve the *TNXB/TNXA* gene [12]. In this case, additional clinical symptoms may arise as a result of *TNX* function impairment, thereby causing the so-called CAH-X syndrome (CAH-X) [19], [20], [21].

Three CAH-X chimeras have been described (CH1, CH2, CH3) based on the location of the junction site (Figure 1B). The CH1 chimera includes the 120 bp deletion of exon 35 (Del120bp) and causes haploinsufficiency [22]. The CH2 chimera is characterized by the alteration (NM_019105.8) c.12174C>G (p.Cys4058Trp) in exon 40 [20]. The CH3 chimera contains the cluster: (NM_019105.8) c.12218G>A (p.Arg4073His), exon 41; c.12514G>A (p.Asp4172Asn) and c.12524G>A (p.Ser4175Asn), exon 43 [13]. The proteins encoded by CAH-X CH2 and CH3 have structural changes that are poorly tolerated due to their negative dominant effect, which express in the form of more severe clinical symptoms [20, 23, 24].

The clinical manifestations of CAH-X include connective tissue disorders, i.e., musculoskeletal (generalized joint hypermobility, subluxations, chronic arthralgias); dermatological (skin hyperextensibility, abnormal scarring); cardiac (congenital defects, atrial–ventricular dilatation); and and/or digestive (herniations, prolapses) disorders [25, 26].

In the most relevant case series published to date, the prevalence of CAH-X is estimated, and its clinical manifestations are described for different populations [13, 19, 22, 24, 27]. In the series where CL forms are highly represented, prevalence reaches 10–15 % [13, 19, 22, 24, 27]. Diagnosis of CAH-X is established based on a clinical approach involving the occurrence of some of its clinical manifestations in a clinical context of CAH. There is not a specific therapy currently available for CAHX syndrome. However, the clinical course of the disease improves with close monitoring and when support and rehabilitation measures are adopted [22].

The objective of this study was to set up a molecular approach to detect all types of CAH-X chimeras and assess their frequency in CAH patients in the Spanish population. Other objectives included examining the distribution of frequencies of the different chimeras to promote screening in these patients to ensure adequate follow-up and clinical interventions, where appropriate.

## Materials and methods

A retrospective, observational, descriptive study was carried out. However, in the light of the rapid advancements in the understanding of CAH-X [15] and the relevance of this entity, it was necessary as well to prospectively examine CAH-X in clinical practice. Our initial sample (n=4,157 index cases) was composed of patients with suspected CAH attended in hospitals in Spain who underwent genetic testing for *CYP21A2* at the Laboratory of Molecular Diagnosis of the HGUGM (2000–2022) and the laboratory of La Paz University Hospital (1995–2000). Based on the molecular data of these CAH patients, we selected patients at risk of suffering CAH-X. Risk was assessed as a function of the presence of at least one allele with a deletion–conversion of the *CYP21A2* gene that could also involve the *TNXB* gene (postgenic or 3′- located junction site). The series includes the whole spectrum of clinical forms of 21OHD: CL (SW, SV), NC, cryptic forms and carriers with functional hyperandrogenism (HF), since all may carry one allele with a *CYP21A2* deletion/conversion. Of the 4,157 index cases, 370 (8.9 %) had at least one allele with deletion–conversion of *CYP21A2*. From those, 184 patients (49.7 %) had an intragenic junction site before *CYP21A2* before exon 6 and thus did not involve the *TNXB* gene and were excluded from the study. The remaining 186 (50.3 %) had a junction site downstream exon 6 and could potentially include *TNXB* variants. Thirty-two affected relatives were also studied testing. A total of 186 families were tested. DNA of the index case was not available in nine families. In six families, we analyzed DNA of nonaffected relatives that secreted the allele under study. CAH patients with deficiencies other than 21OHD were excluded from the study. The study was approved by the CEIm of the HGUGM. The study was conducted in compliance with the tenets of the 1964 Declaration of Helsinki and subsequent updates.

Molecular analysis of *CYP21A2* was based on gene-specific PCR amplifications followed by techniques such as specific allele hybridization (allele-specific oligonucleotide, ASO) Sanger sequencing and SnaPshot; MLPA gene dosage was performed on genomic DNA samples, as described by Ezquieta [28–33] and reviewed by Arriba & Ezquieta [34].

Molecular analysis of *TNXB* was based on different approaches implemented for the purposes of this study (Supplementary Table 1). The CAH-X CH1 chimera was detected using two approaches: (1) gene dosage analysis by MLPA using the CAH sample analysis kit (SALSA MLPA Probemix P050 CAH version C1 or D1 (MRC Holland, Amsterdam, Netherlands) following manufacturer’s instructions, verified against the original technique of reference (Southern TaqI y BglII sonda pC21/3c). Analysis was performed using the Coffalyzer (Coffalyser.Net™, MRC Holland, Ámsterdam, Netherlands) software package; (2) screening by capillary gel electrophoresis of the PCR *TNXB* fragment (*TNXB* ex33s-ex37as amplicon, see Supplementary Table 1) to detect the presence/absence of heteroduplex, which are suggestive of the presence of Del120pb, characteristic of the CH1 chimera. For confirmation, we performed the sequencing of the exon 35 obtained from nested PCR with the primer 35s (5′-TCATCGCCTCGCATTTCCTCTC-3′) designed in the laboratory; and MLPA analyses. For the detection of the CH2 and CH3 chimeras, gene-specific PCR amplification of the *TNXB* gene was performed (Supplementary Table 1) by sequencing exons 40, 41, and 43 of *TNXB* using fluorescent dideoxynucleotides (BigDyeTM Terminator v3.1 Cycle Sequencing Kit, Applied Biosystems by Thermo-Fisher Scientific^®^, Waltham, MA, USA). The primers used for the gene-specific PCR and sequencing of *TNXB* exons are described elsewhere by Gao et al. [22]. Exon 40 sequencing was performed using the *TNXB38F* primer described by Rymen et al. [35]. Figure 1C and D displays the amplification strategies and sequencing primers used for the CAH-X chimeras.

All PCRs were performed on a ProFlexTM PCR thermal cycler (Applied Biosystem^®^, Waltham, MA, USA). Purification of the amplicons obtained was carried out with the QIAquick^®^ PCR Purification Kit (QIAGEN GmbH, Hilden, Germany). Amplicon sequencing and sample processing for MLPA were performed on the ABI PRISM 3730xl genetic analyzer (Applied Biosystem^®^, Waltham, MA, USA) of the HGUGM Unit of Genomics. The analysis of the sequences obtained was performed with Chromas and Sequence Scanner Software 2.0 (Applied Biosystem^®^, Waltham, MA, USA).

The results for carriers of CAH-X chimeras were reported to the responsible study physicians in the tertiary university hospitals La Paz, Virgen de la Arrixaca and Marqués de Valdecilla. The responsible physicians selected the most feasible type of clinical evaluation (performing a review of the medical history of all patients, face-to-face consultations, where possible, telephone consultations, and/or complementary tests, some of which are still pending). It is worth noting that the study was carried out during the COVID pandemic, when the presence of patients and relatives in hospitals was restricted. The clinical manifestations detected were recorded in a table designed *ad hoc* on an Excel spreadsheet, following the model designed by Miller and Merke [25].

A descriptive statistical analysis was conducted. For quantitative and qualitative variables, frequencies and percentages were calculated. Hypothesis testing was performed to compare the results of our study with the ones obtained in previous studies. A p-value<0.05 was considered statistically significant.

## Results

### Frequency and distribution of CAH-X chimeras in the Spanish population

**Figure 2:**
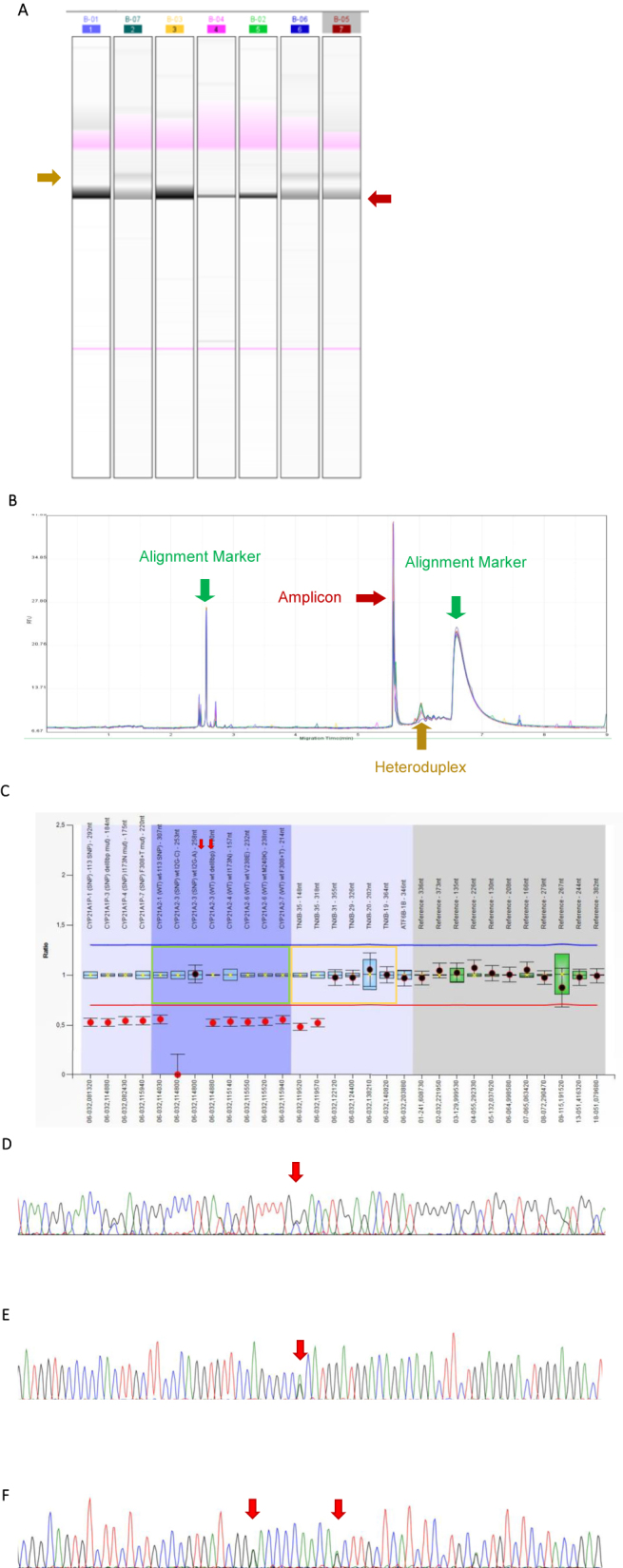
Molecular strategy for CAH-X quimeras. (A–B) Capillary gel electrophoresis. (A) Example of a run with several positive samples (tracks 2, 6, and 7), which exhibit the heteroduplex band (indicated in mustard color). The remainder of samples was negative for Del120pb. (B) Profile of the chromatogram obtained when screening was positive for heteroduplex. The peaks corresponding to alignment markers (in green), the amplicon (in maroon), and the resulting heteroduplex (in mustard) are indicated. (C) MLPA results: visualization of the results obtained for a Del120pb carrier (exon 35 *TNXB*). In green: probes corresponding to the *CYP21A2* gene. In yellow: probes corresponding to the *TNXB* gene. Red arrows indicate the probes that hybridize against exon 35 of the *TNXB* gene. (D–F) Sequences corresponding to the regions containing SCAH-X characteristic variants. (D) Exon 40 of *TNXB*, where position c.12174C>G (p.Cys4058Trp) is indicated with an arrow, corresponding to a patient who was heterozygous for the variant analyzed. (E) Exon 41 of the *TNXB* gene. The arrow indicates position c.12218G>A (p.Arg4073His), which is characteristic of the CAH-X CH3 chimera. (F) Region corresponding to exon 43 of the *TNXB* gene. The arrows indicate positions c.12514G>A (p.Asp4172Asn) and c.12524G>A (p.Ser4175Asn), respectively (the two were heterozygous for the analyzed variants).

The optimized approach to detect the CAH-X chimeras, CH1, CH2, and CH3, is described on Supplementary Table 1. The characteristics of the patients who underwent molecular analysis (186 patients, see Materials and methods) are shown in Table 1. Figure 2 contains some examples of the results obtained with the different techniques.

**Table 1:** Patient characteristics.

	Men (n=65)	Women (n=121)
Age, mean, years	25.7 (0.6–55.3)	23.8 (2.5–58.9)
CAH forms, n		
SW	41	33
SV	5	12
NC	17	65
FH	2	11

SW, salt-wasting classic form; SV, simple virilizing classic form; NC, nonclassic forms of CAH; FH, functional hyperandrogenism; CAH, congenital adrenal hyperplasia.

Table 2 shows the results for our series and previous series. Seventy-eight patients were carriers of one or several CAH-X chimeras (41.9 %). With regard to the CAH-X CH1 chimera, analysis of 73 clinical samples by MLPA containing *TNXB* probes (Materials and methods) enabled the identification of 30 CH1 patients. Retrospective heteroduplex screening of 84 patients identified 16 additional cases, which were confirmed by MLPA. In total, 46 patients were carriers of the CH-1 chimera (24.7 %, 46/186); 24 had the CH-2 chimera (12.9 %, 24/186); and 8 were carriers of the CH-3 chimera (4.3 %, 8/186). Familial segregation was confirmed in 21 CAH-unaffected relatives who were carriers of the CAH-X alleles.

**Table 2:** Data from SCAH-X prevalence studies for different cohorts.

Study	Lao et al. [27]	Gao et al. [22]	Marino et al. [13]	Paragliola et al. [19]
Analyzed CAH patients^a^, n	135	424	337	196
Clinical forms, n	N.A.	SW 137 SV 249 NC 38	N.A.	SW 99 SV 42 NC 55
DelB^b^ patients, n (%)	72 (29)	94 (22)	66 (19.5)	74 (37.8)
SCAH-X patients^c^, n	21	59	48	21
SCAH-X prevalence for the total of patients, %	15.6	13.9	14.2	10.7
SCAH-X prevalence for DelB patients, %	29	62.8	72.7	28.4
CAH-X CH1, n (%)	13 (18.1)	35 (59)	26 (54.2)	13 (61.9)
CAH-X CH2, n (%)	8 (11.1)	13 (22)	21 (43.8)	7 (33.3)
CAH-X CH3, n (%)	0	11 (18.6)	1 (0.9)	1 (4.8)

^a^Number of index cases. ^b^Deletion in *CYP21A2*, considering different junction sites (intragenic and 3′- or postgenic). ^c^Includes monoallelic and biallelic patients. DelB, deletion–conversion in *CYP21A2*; CAH, congenital adrenal hyperplasia; NC, nonclassic forms of CAH; N.A. not available; SW, salt-wasting classic form; CAH-X, CAH-X syndrome; SV, simple virilizing classic form.

In our cohort, we found two patients who were homozygous for the classic form of CAH-X: a case of CAH-X CH1 (CH1/CH1) and another of CAH-X CH2 (CH2/CH2).

**Figure 3:**
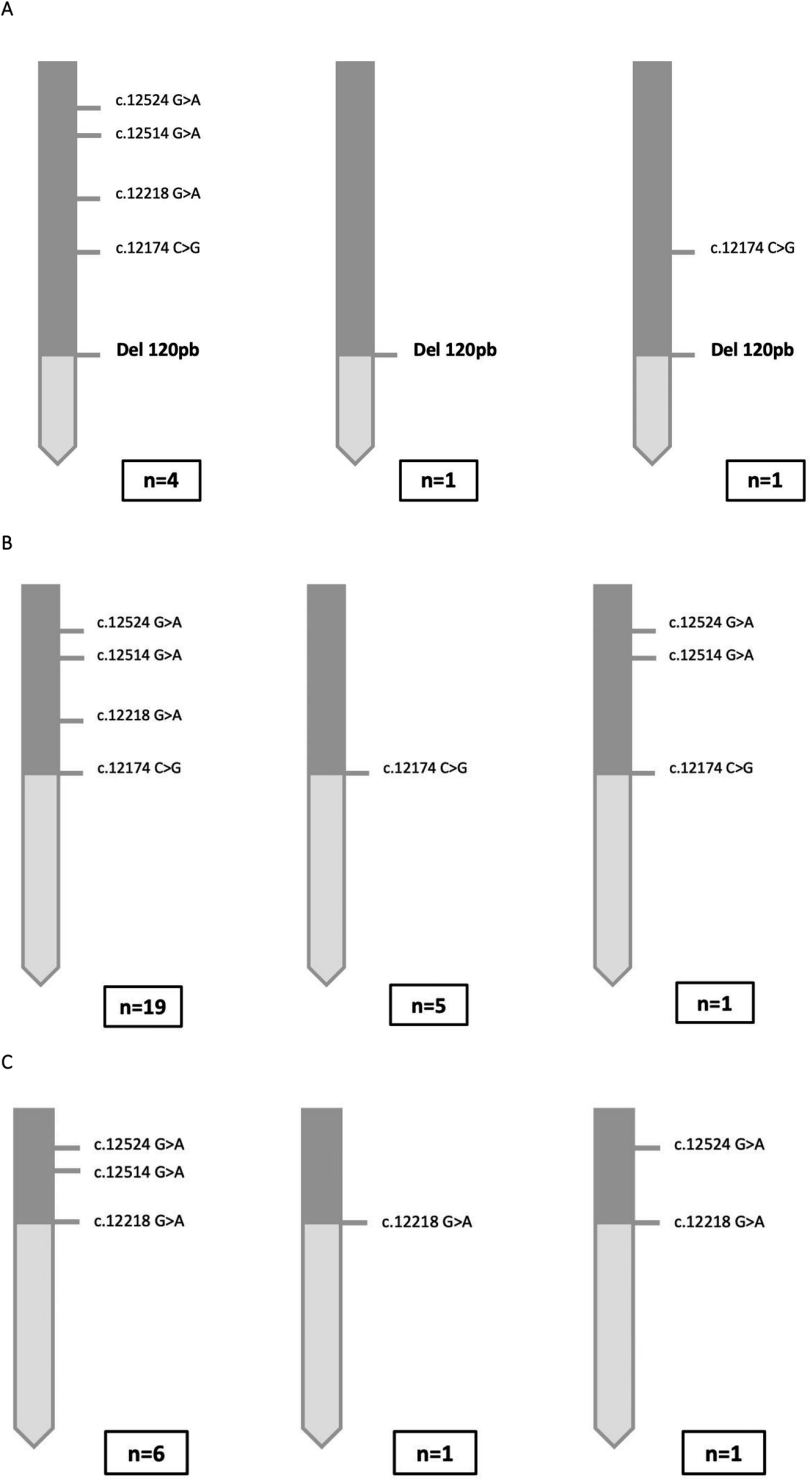
Haplotypes with the different variants of the CAH-X chimeras found in Spanish patients. (A) Variants of the CAH-X CH1 chimera. The most frequent allele was the one that harbored Del120pb, along with another four variants. An allele only included Del120pb. Another allele harbored Del120pb and the c.12174C>G variant (p.Cys4058Trp). In the case of the CAH-X chimera, complete molecular analysis was performed of only six alleles. The reason is that, in the case of these chimeras, once the presence of Del120pb was detected by capillary gel electrophoresis/MLPA, the molecular study of exons 40, 41, and 43 was not performed. (B) The most frequent variant of the CAH-X CH2 chimera was the one that harbored the c.12174C>G mutation (p.Cys4058Trp), along with the molecular abnormalities present in exons 41 and 43. The second most frequent variant was the one that only included the abnormality that is characteristic of this chimera. (C) As it occurs with the other two chimeras, the most frequent variant of the CAH-X CH3 chimera was the one that included the cluster that characterizes it. Another two types of variants were identified: a variant that only contained an abnormality in exon 41 c.12218G>A (p.Arg4073His) and another variant that had an abnormality in exon 41 and one of the occasional abnormalities of exon 43 c.12524G>A (p.Ser4175Asn).

Several variants of the CAH-X chimeras were identified (Figure 3). In all chimeras, the most frequent type was the one that contained all *TNXA* variants until the location of the junction site.

### Comparative analysis of frequencies with other populations

The frequencies of the different CAH-X chimeras should be assessed in case series of patients having the same clinical form of the disease, since the frequency of alleles containing deletions/conversions varies across the different clinical forms. Thus, 25 % of patients with the CL form have alleles containing deletions/conversions [28], as compared to 5 % for the NC forms [36] and 1 % for HF carriers [36] in the Spanish population. The results of comparative analysis will be more accurate if only CAH carriers of chimeric alleles are included (whatever the clinical form is). Table 2 shows that the frequencies of the CAH-X alleles in our population (21.1 %; 78/370) are consistent with the ones reported by Lao & Merke 2019 (29 %; 21/72) [27] and Concolino 2022 (28.4 %; 21/74) [19]. In contrast, the Gao [22] and Marino [13] series shows substantially higher frequencies of 62.8 % (59/94) and 72.7 % (48/66) (p<0.0001), respectively. This discrepancy could be explained by the heterogeneous composition of the series, in terms of junction sites of the chimeras. If we exclude the chimeras with an intragenic junction site that locates before exon 6 (which cannot involve *TNXB*), the frequency of CAH-X chimeras is 41 %. This frequency cannot be calculated for other series, since a molecular definition of these specific cases was not provided.

No differences were observed in the distribution of frequencies of the different types of chimeras. The CAH-X CH1 chimera was the most frequent in all series, whereas CH3 was less frequently found.

### Clinical pattern of occurrence

Twenty index cases have been evaluated clinically to date (Hospital Universitario La Paz, Hospital Clínico Universitario Virgen de la Arrixaca y Hospital Universitario Marqués de Valdecilla). Five in twenty patients were carriers of the CH1 chimera, five were carriers of the CH2 chimera, and 10 did not show any *TNXB* abnormality. Among the carriers of some CAH-X allele, seven (7/10 70 %) exhibited clinical manifestations, of whom 4/5 patients were carriers of the CH1 chimera and 3/5 of CH2 (Table 3).

**Table 3:** Clinical findings in CAH patients with CAH-X chimeras who underwent clinical evaluation in the following hospitals: La Paz University Hospital, Marqués de Valdecilla Hospital and Virgen de la Arrixaca Hospital.

Patient	Gender	CAH phenotype	Musculo-skeletal manifestations	Cutaneous manifestations	Gastrointestinal manifestations	Cardiac manifestations	Other
**CAH-X CH1**							

P-004	F	SW		Haematomas			
P-053	M	NC					
P-098	F	NC	Dislocations	Cutaneous hypersensitivity			
P-134	M	FH	Dislocations, chronic arthralgia				
P-154	F	NC		Hypertrophic scar after laparoscopy (cholecystectomy), haematomas in the lower limbs		Normal cardiological evaluation	Pes planus in childhood

**SCAH-X CH2**							

P-032	F	FH					Perthes disease, Giand lung bulla
P-041	M	SW	Chronic arthralgia, dislocations, tendinitis/bursitis/fasciitis		Colitis/proctitis		
P-062	M	SW	Tendonitis/bursitis/fasciitis				
P-074	F	NC					
P-144	F	SV	Chronic arthralgia (fingers, knuckles, wrists and in the neck), mild scoliosis, extensor pollicis brevis tendonitis)	Frequent hematomascol		Normal cardiological evaluation	

F, female gender; FH, functional hyperandrogenism; CAH, congenital adrenal hyperplasia; M, male gender; NC, non-classic forms of CAH; SW, classic salt-wasting form; CAH-X, SCAH-X syndrome; SV, classic simple-virilizing form.

## Discussion

Defining the new CAH-X syndrome that some CAH patients may progressively develop represents a challenge to the physicians involved in the follow-up of this frequent congenital disease. For an optimal follow-up, the clinical signs that characterize CAH-X [15], which differ from those of CAH, should be searched for in every CAH patient. The molecular identification of carriers of CAH-X chimeric alleles (involving the two genes *CYP21A2* and *TNXB*) will make it possible to adopt a more accurate follow-up approach focused in those patients. The screening protocol suggested in this study enables the detection and identification of the *TNXB* variants that are characteristic of CAH-X chimeras. The MLPA technique used for *CYP21A2* gene dosage analysis in molecular diagnosis of CAH now enables routine screening for CAH-X. However, MLPA only detects the CAH-X CH1 chimera, which is the most frequent one, but could not be the chimera causing the most severe forms of CAH-X [15]. It is necessary that screening for CH2 and CH3 chimeras is performed, as the one proposed in this study. In the light that these chimeras were not identified by previous techniques, retrospective studies are necessary. These studies would benefit from the heterodimer screening protocol described here, which detects CH1.

To the best of our knowledge, this is the largest study involving patients with all the clinical forms of CAH. This study did not only include the severe forms of CAH (SW and SV) but also the numerous forms that may carry a severe deletion/conversion allele (NC, cryptic and even monoallelic forms, i.e., carriers with FH). To optimize diagnostic yield through molecular selection, screening was targeted to CAH carriers of the *CYP21A2* alleles that may involve the *TNXB* gene. Otherwise said, clinical evaluation of SED signs was directed to carriers of large deletions/conversions with a junction site in region 3′- or postgenic involving both *CYP21A2* and *TNXB* genes.

In previous studies on the prevalence of CAH-X, a more limited clinical spectrum of the disease was analyzed. Thus, early studies only analyzed the CL forms [23], while a definition was not always provided for this form [13, 27]. It is only in the most recent studies that the clinical forms, including mild forms of this deficiency, have been included and defined [19, 22]. As observed by Concolino [15], the definition provided for “30-kb deletion” is not comparable across studies. These facts explain discordance in the CAH-X frequencies obtained across studies (Table 2).

A prevalence of CAH-X as low as 1.9 % could be initially striking (Table 2). However, similar frequencies are found in the comparative analysis of data for CAH patients with deletions/conversions (Table 2), namely: 21.1 , 29, and 28.4 % in the studies conducted by Lao, Merke, and Concolino, respectively [19, 27]. In contrast, substantially higher frequencies are reported by authors such as Gao [22] and Marino [13]. This high frequency could be explained by a larger representation of *CYP21A2* chimeras with a junction site on 3′ in these series. Different allele frequencies have been observed in CAH patients across different geographical regions by preferential dissemination. This difference is attributed to the founder effect, genetic drift, and an evolutionary advantage of carriers [37–40].

In terms of clinical patterns of occurrence, this preliminary study was carried out in three tertiary hospitals located in distant regions of the country (Cantabria, Madrid, and Murcia). We noticed that a large proportion of patients (although not all CAH-X allele carriers) exhibited clinical manifestations associated with EDS, being musculoskeletal disorders the most frequent. In our cohort, limited clinical data are available in terms of number and thoroughness of evaluation (complementary studies not performed, evaluation limited to a review of the medical history without a targeted evaluation of EDS). Unfortunately, none of the two patients who were homozygous for CAH-X underwent clinical evaluation. The potential genotype/phenotype relationship between severity and type of chimera and homozygosity or heterozygosity has not yet been deeply explored, but more severe forms seem to be associated to the biallelic forms of CAH-X.

The number of cases reported to date is very limited. Also, a clinical evaluation was not performed in all patients who underwent molecular testing or in carriers of CH2 and CH3 chimeras. The prognosis of these patients would certainly improve with close follow-up and adequate clinical interventions. Definitive conclusions cannot be drawn due to the limited number of cases reported and short time from disease onset analyzed.

This is the first study to assess the prevalence of CAH-X in the Spanish population. In this study, we developed and validated a screening protocol for all known types of CAH-X–causing chimeras. This protocol can be used in any basic genetic testing laboratory. A clinical evaluation of CAH-X carriers is currently being performed. However, we are faced with some limitations: firstly, a clinical evaluation could not be performed of all CAH-X carriers identified. Secondly, we could only perform a review of medical records due to the restrictions imposed by the SARS-COV-2 pandemic. In addition, patients are not evaluated by the same physician, since they are treated in hospitals from different regions of Spain. A clinical evaluation was not performed of non–CAH-X CAH patients, although 27 % have been reported to develop signs of EDS [22]. Patient selection is not exempt from subjectivity, as EDS signs were evaluated in previously identified carriers of CAH-X chimeras. Last, but not least, a high proportion of patients are still pediatric patients. Since the onset of clinical signs occurs progressively and is age dependent, pediatric patients may have not yet experienced the clinical manifestations of EDS.

Despite recent advances, our understanding of this new syndrome is still limited. Further studies are necessary to assess its impact on different populations of CAH patients. It is essential to determine whether its pattern of occurrence, being a dominant monoallelic pattern, manifests in carriers and mild forms of this deficiency or only in the CL forms. Given the frequency of CAH and its chimera-carrying alleles, which may involve the *CYP21A2* and *TNXB* genes, early diagnosis of CAH-X is recommended to prevent and manage clinical manifestations in carriers of these alleles.

## Supplementary Material

Supplementary Material
