# Supplementary material for: Molecular characterization of the new clinical entity associated with congenital adrenal hyperplasia: the CAH-X syndrome in the Spanish population
Source: Adv Lab Med. 2023 Aug 25;4(3):258–67. doi: 10.1515/almed-2023-0071 (PMC10701499; doi:10.1515/almed-2023-0071)
Supplement: Supplementary file 1 — Supplementary Material [file j_almed-2023-0071_suppl_001.doc]

**Supplementary material:**

**Complementary Table 1.** Reagents used for the different gene-specific PCRs performed for the molecular characterization of CAH-X chimeras, and testing conditions.

|  | |  |  |
| --- | --- | --- | --- |
| **Micale, 2019 - 1º PCR** | V (uL) | PCR amplification conditions | |
| Long-PCR *TNXB*ex31 F | 0,5 |  | Initial denaturalization 95°C – 3min  Denaturalization 95°C – 30s  Annealing 62°C – 30s  Extension 72°C – 7min  72°C – 7min |
| Long-PCR *TNXB*ex44 R | 0,5 |  |
| dNTPs (Deoxynucleoside Triphosphate Set PCR Grade, Roche Diagnostics GmbH, Mannheim, Germany) | 1 | 30 cycles |
| Expand High Fidelity Buffer, with 15 mM MgCl2 10x concentrated (Roche Diagnostics Deutschland GmbH, Mannheim, Germany) | 5 |
| Water for injectable preparations (B. Braun, Melsungen, Germany) | 41 |
| Expand™ High Fidelity PCR System (Roche Diagnostics Deutschland GmbH, Mannheim, Germany) | 0,5 |  |
| DNA (100 ng/uL) | 2 |  |
| **Micale, 2019 – 2º PCR** | V (uL) |  | PCR amplification conditions |
| *TNXB* ex33s | 1 |  | Initial denaturalization 95°C – 3min  Denaturalization 95°C – 30s  Annealing 58°C – 20s  Extension 72°C – 1min  72°C – 7min |
| *TNXB* ex37(i)as | 1 |  |
| dNTPs (Deoxynucleoside Triphosphate Set PCR Grade, Roche Diagnostics GmbH, Mannheim, Germany) | 1 | 32 cycles |
| PCR Buffer with MgCl2 10x concentrated (Roche Diagnostics Deutschland GmbH, Mannheim, Germany) | 5 |
| Water for injectable preparations (B. Braun, Melsungen, Germany) | 40 |
| Taq ADN Polymerase, 5 U**/**μ**l** (Roche Diagnostics Deutschland GmbH, Mannheim, Germany) | 0,5 |
| DNA (dilution 1:100 amplicon 1st PCR) | 1,5 |  |
| **Gao, 2020** | V (uL) |  | PCR amplification conditions |
| *TNXB* ex35(Del120pb) F | 0,5 |  | Initial denaturalization 98°C – 3min  Denaturalization 95°C – 30s  Annealing 63°C – 30s  Extension 72°C – 3min  72°C – 10min |
| *TNXB* 3’UTR R | 0,5 |  |
| dNTPs (Deoxynucleoside Triphosphate Set PCR Grade, Roche Diagnostics GmbH, Mannheim, Germany) | 0,5 | 40 cycles |
| HF Phusion 5X buffer (Thermo Fisher Scientific Inc., Waltham, Massachusetts, USA) | 5 |
| Water for injectable preparations (B. Braun, Melsungen, Germany) | 22 |
| Phusion™ High-Fidelity ADN Polymerase (Thermo Fisher Scientific Inc., Waltham, Massachusetts, USA) | 0,3 |
| DNA (100 ng/uL) | 1,5 |  |

DNA, deoxyribonucleic acid; as, *antisense;* dNTPS, *deoxynucleotide triphosphates*; F, *forward*; PCR, *polymerase chain reaction*; R, *reverse*; s, *sense*; Taq, Taq polymerase; UTR, *untraslated region;* V, volume.
